# High-Sensitivity C-reactive Protein as a Predictive Biomarker of Coronary Artery Disease Severity: A Cross-Sectional Study in Bangladesh

**DOI:** 10.7759/cureus.107695

**Published:** 2026-04-25

**Authors:** Md. Maruf-Ur- Rahman, Md. Rafiqul Islam, Wazeda Begum, Md. Musaddequl Alam, Md. Fakhrul Islam Khaled, A. H. M. Golam Kibria

**Affiliations:** 1 Biochemistry, Dhaka National Medical College, Dhaka, BGD; 2 Biochemistry and Molecular Biology, Jagannath University, Dhaka, BGD; 3 Dermatology and Venereology, Dhaka National Medical College, Dhaka, BGD; 4 Cardiology, Dhaka National Medical College, Dhaka, BGD; 5 Cardiology, Bangladesh Medical University (BMU), Dhaka, BGD; 6 Epidemiology and Biostatistics, Centre for Medical Research and Development (CMRD), Dhaka, BGD

**Keywords:** coronary artery disease (cad), high-sensitivity c-reactive protein (hs-crp), multivessel cad, prediction, sensitivity, specificity

## Abstract

Background

Bangladesh bears a substantial burden of advanced coronary artery disease (CAD), yet affordable biomarkers that can help predict angiographic disease severity before invasive assessment remain limited. High-sensitivity C-reactive protein (hs-CRP), a marker of vascular inflammation, has been linked to atherosclerosis, but its utility in predicting multivessel disease in resource-limited settings remains uncertain. The primary objective of this study was to compare hs-CRP levels between patients with angiographically confirmed CAD and those without CAD. The secondary objective was to evaluate the association and predictive utility of hs-CRP with the angiographic severity of CAD, with particular emphasis on multivessel involvement among patients undergoing coronary angiography.

Methods

This cross-sectional study included 300 adult participants undergoing coronary angiography, comprising 150 angiographically confirmed CAD cases and 150 controls without CAD. Participants were enrolled purposively from the Department of Cardiology of Bangladesh Medical University (BMU) and Dhaka National Medical Institute Hospital (DNMIH), Dhaka, Bangladesh, from January 2024 to December 2024. CAD severity was categorised as single-vessel, double-vessel, and triple-vessel disease according to the number of major coronary arteries involved. Laboratory investigations, including fasting lipid profile, glycosylated haemoglobin (HbA1c), and hs-CRP, were measured using standardised automated laboratory platforms. Ethical approval was obtained from the Institutional Ethical Review Board of Dhaka National Medical College, Dhaka.

Results

Most participants in both groups were aged 41-60 years, and the median age was similar between cases and controls (52 (19-68) years vs. 52 (28-70) years, p-value: 0.569). Male participants were significantly more frequent in the case group than in the control group (114 (76.0%) vs. 95 (63.3%), p-value: 0.017). hs-CRP levels were significantly higher among CAD cases than controls (p value: <0.001) and increased progressively with angiographic severity. A moderate positive correlation was observed between hs-CRP and the number of diseased vessels (rs=0.449, p value:<0.001). Receiver operating characteristic (ROC) analysis showed moderate ability of hs-CRP to discriminate multivessel CAD (area under the curve (AUC)=0.729, 95% CI: 0.651-0.808). A cut-off value of ≥22.89 mg/L provided 95.1% specificity and 97.0% positive predictive value. Triple-vessel disease was present in 68 (45.3%) cases, while hypertension and diabetes were the most common comorbidities.

Conclusion

Elevated hs-CRP was significantly associated with both the presence and angiographic severity of CAD. It may serve as a useful adjunctive biomarker for identifying advanced coronary disease in resource-limited settings.

## Introduction

Coronary artery disease (CAD) persists as a significant global health issue and is among the foremost causes of mortality and disability globally, especially in low- and middle-income nations where cardiovascular risk factors are escalating rapidly. In 2019, the global burden of disease (GBD) data indicated that CAD accounted for 16.2% of all-cause mortality and 7.19% of disability-adjusted life years (DALY) globally [1].

Evidence further indicates that South Asian populations experience earlier onset and more aggressive forms of CAD than their Western counterparts, a pattern that in Bangladesh is likely driven by the convergence of unhealthy dietary practices, physical inactivity, widespread tobacco use, and limited access to timely preventive and curative healthcare services, factors that together continue to fuel the growing national burden of this disease [2]. Despite advancements in diagnostic and therapeutic approaches, the prevalence of CAD continues to rise, with a substantial proportion of patients presenting with advanced coronary involvement or multivessel CAD at first evaluation. Multivessel CAD is associated with a worse prognosis, increased complications, prolonged hospital stays, and elevated in-hospital mortality relative to single-vessel disease, underscoring the significance of early disease extent recognition in clinical risk differentiation and therapeutic regimen selection [3,4]. While coronary angiography is the definitive method for detecting CAD and evaluating stenosis severity, the inequitable allocation of medical facilities in Bangladesh may impede prompt testing. Advanced/multivessel CAD is primarily predicted by a cluster of traditional risk factors: age, diabetes, hypertension, atherogenic lipids, renal impairment, and smoking, with newer markers (triglyceride-glucose index (TyG), C-reactive protein-to-albumin ratio (CAR), high-sensitivity C-reactive protein (hs-CRP), epicardial fat, and high coronary artery calcium score (CAC)) refining risk stratification, especially in acute coronary syndrome (ACS) and high-risk stable patients [3,5,6].

A growing body of evidence has highlighted the central role of inflammation in the pathogenesis and progression of atherosclerosis. hs-CRP is an acute phase reactant and a calcium-binding pentameric protein composed of five identical non-covalently linked 23 kDa subunits synthesized in the liver in response to interleukin 6, interleukin 1, and tumor necrosis factor alpha [7]. It is one of the most extensively investigated and clinically validated biomarkers of inflammation in cardiovascular disease. In angiographic research, hs-CRP is preferred over standard CRP because its superior analytical sensitivity allows detection of chronic low-grade systemic inflammation [8]. In addition to reflecting inflammatory activity, accumulating evidence indicates that hs-CRP may actively contribute to atherogenesis through mechanisms such as endothelial dysfunction, upregulation of adhesion molecules, increased oxidative stress, and promotion of a prothrombotic state [9]. Elevated hs-CRP levels have consistently been associated with endothelial impairment, plaque vulnerability, and adverse cardiovascular outcomes, supporting its role as a robust biomarker of low-grade systemic inflammation in CAD [10].

Prior studies have yielded inconsistent findings, have often focused on long-term outcomes rather than angiographic extent, or have been conducted in populations that may not reflect contemporary practice in South Asian or resource-constrained settings. Previous studies examined the associations between hs-CRP and the presence of CAD and angiographic CAD severity. However, angiographic CAD severity has reported inconsistent findings, with some demonstrating strong correlations, while others found weak or no associations [11-13]. Consequently, there is a need for context-specific data evaluating whether hs-CRP can serve as a reliable marker for predicting multivessel CAD before invasive assessment. However, despite its established prognostic significance, uncertainty remains regarding the ability of hs-CRP to discriminate the severity of CAD, particularly in differentiating patients with single-vessel involvement from those with multivessel disease. Hs-CRP is an important biomarker indicating persistent inflammatory risk in coronary atherosclerosis. Integrating hs-CRP into standard clinical practice may improve cardiovascular risk assessment and inform treatment choices. Because the assay is inexpensive, widely available, and easily incorporated into routine laboratory testing, hs-CRP has attracted considerable interest as a potential tool for refining cardiovascular risk assessment beyond traditional factors.

In this context, the primary objective of this study was to compare hs-CRP levels between patients with angiographically confirmed CAD and those without CAD. The secondary objective was to examine the association between hs-CRP levels and the angiographic severity of CAD and to evaluate its potential utility in predicting the extent of disease, particularly the presence of multivessel coronary involvement among patients undergoing coronary angiography.

## Materials and methods

Study design and setting

A cross-sectional analytical study was conducted in the Department of Cardiology of Bangladesh Medical University (BMU) in collaboration with the Department of Cardiology, Dhaka National Medical Institute Hospital (DNMIH), Dhaka, Bangladesh, from January 2024 to December 2024. Ethical approval was obtained from the Institutional Ethical Review Board (IERB) of Dhaka National Medical College, Dhaka (Reference Number: DNMC/IERB/Ethical/2023/2027). Written informed consent was taken from all participants after explaining the aims and objectives of the study, and confidentiality of personal information was ensured throughout the research study in accordance with the Declaration of Helsinki.

Study population and sampling

A total of 300 adult participants were enrolled using a purposive sampling technique from patients undergoing coronary evaluation by angiography, including 150 cases with CAD and 150 controls without CAD. Patients of both sexes aged between 18 and 70 years who were referred for diagnostic coronary angiography from inpatient wards, outpatient cardiology clinics, and emergency services were initially screened for eligibility. Individuals who fulfilled the predefined inclusion and exclusion criteria were identified at the time of angiographic evaluation. Eligible participants were enrolled sequentially as they became available, and recruitment continued until the required sample size for both case and control groups was achieved.

Eligibility criteria

Patients were included in the case group if they had angiographically or clinically confirmed coronary artery disease according to World Health Organization (WHO) and European Society of Cardiology (ESC) guidelines [14], while individuals with the absence of CAD under a coronary angiogram served as the control group. CAD was defined as the presence of luminal stenosis greater than 50% in at least one major epicardial coronary artery on coronary angiography. All angiographic assessments were performed and verified by a trained cardiologist to confirm the presence of CAD for case identification, differentiate participants for appropriate control group enrollment, and accurately determine the extent of vessel involvement. Patients were excluded from the case group if they had acute or chronic inflammatory diseases, congenital heart disease, chronic liver disease, chronic lung disease, malabsorption syndromes, a history of solid organ transplantation, known primary hyperparathyroidism or malignancy, advanced chronic kidney disease, or were receiving medications known to influence inflammatory or metabolic parameters, including bisphosphonates, anticonvulsants, antipsychotics, or antidepressants.

Controls were required to have no prior history of cerebrovascular disease, or peripheral arterial disease, chronic kidney or liver disease, inflammatory or autoimmune disorders, acute infection within the preceding four weeks, malignancy, pregnancy, or be taking corticosteroids, statins, immunosuppressive agents, or psychotropic medications that could alter inflammatory markers or lipid metabolism.

Data collection and study variables

Data was collected using a predesigned structured case record form. Sociodemographic variables included age, sex, residence, educational status, and socioeconomic background. Clinical data comprised smoking status, body mass index (BMI), history of diabetes mellitus and hypertension, and family history of coronary artery disease. For patients with coronary artery disease, angiographic findings were recorded, including the number of vessels involved, categorized as single-vessel disease, double-vessel disease, or triple-vessel disease, and further classified into single-vessel or multivessel disease. Laboratory variables included glycosylated hemoglobin (HbA1c), fasting lipid profile, and inflammatory markers, including hs-CRP.

Sample collection and laboratory analysis

After at least 10 hours of overnight fasting, approximately 3.0 mL of venous blood was drawn into sterile ethylenediaminetetraacetic acid (EDTA)-containing tubes and stored at −80°C. An additional 3.0 mL of venous blood was collected into plain vacuum tubes for serum separation. Serum samples were centrifuged and analysed on the same day or stored appropriately until processing. Biochemical parameters, including HbA1c, fasting lipid profile, and CRP, were measured using standard enzymatic methods. All laboratory analyses were performed using automated platforms, including the VITROS® 5600 Integrated System (Ortho Clinical Diagnostics, Raritan, NJ, USA), ADVIA Centaur XP Immunoassay System (Siemens Healthineers, Tarrytown, NY, USA), and Atellica Solutions CH 930 (Siemens Healthineers), following manufacturer protocols and routine internal quality-control procedures. hs-CRP was measured using the Atellica CH analyzer (Siemens Healthineers) using a latex-enhanced immunoturbidimetric method, with an analytical measuring range of 0.16-200 mg/L.

Statistical analysis

Data were analysed using IBM SPSS Statistics software, version 26 (IBM Corp, Armonk, NY, USA). The normality of continuous variables was assessed using the Shapiro-Wilk test. Normally distributed data were presented as mean ± standard deviation (SD), whereas non-normally distributed data were expressed as median (range). Categorical variables were summarised as frequencies and percentages. Comparisons between case and control groups were performed using the independent-samples t-test for normally distributed continuous variables and the Mann-Whitney U test for non-normally distributed variables. Categorical variables were compared using the Chi-square test or Fisher’s exact test, as appropriate. For comparisons across coronary artery disease subgroups based on angiographic severity, the Kruskal-Wallis test was used for continuous variables, followed by post-hoc pairwise comparisons when indicated. Associations between hs-CRP levels and the extent of coronary vessel involvement were evaluated using Spearman’s rank correlation coefficient. Receiver operating characteristic (ROC) curve analysis was performed to assess the diagnostic performance of hs-CRP in predicting multi-vessel coronary artery disease. The area under the curve (AUC) with 95% confidence intervals (CIs) was calculated. The optimal cut-off value was determined using Youden’s index, and corresponding sensitivity, specificity, positive predictive value, negative predictive value, and overall diagnostic accuracy were reported. Missing data were minimal and managed using complete-case analysis. Outliers were assessed using graphical methods, including boxplots and distribution inspection, and were retained if considered clinically plausible. All statistical tests were two-tailed, and a p-value of <0.05 was considered statistically significant.

## Results

The demographic characteristics of the study participants demonstrated some notable differences between the case and control groups. The age distribution was comparable between groups across all age categories, with the majority of participants in both groups falling within the 41-60-year range. No statistically significant difference was observed in median age between cases and controls (52 (19-68) years vs. 52 (28-70) years, p-value: 0.569), indicating that the two groups were age-matched. In terms of gender distribution, males constituted a significantly higher proportion in the case group compared to the control group (114 (76.0%) vs. 95 (63.3%), p-value: 0.017). BMI also showed statistically significant variation, with overweight status being more prevalent among cases (115 (76.7%)) than controls (80 (53.3%)), while normal BMI was more frequent in controls (58 (38.7%)) compared to cases (16 (10.7%)). The average BMI was significantly higher in the case group compared to the control group (27.25 (23.20-33.30) kg/m^2^ vs. 25.70 (22.90-32.60) kg/m^2^, p-value: < 0.001). These findings suggest that gender and BMI differed significantly between groups, whereas age distribution remained comparable (Table 1).

**Table 1:** Distribution of the participants according to their demographic characteristics (N=300) ᵃ Chi-square test, ᵇ Mann-Whitney U test was done; Data are presented as frequency (%) and median (range)

Demographic characteristics		Participants		Total	p-value
		Case	Control		
		n=150	n=150		
Age (years)	18-30	1 (0.7)	3 (2.0)	4 (1.3)	ᵃ0.499
	31-40	7 (4.7)	10 (6.7)	17 (5.7)	
	41-50	64 (42.7)	52 (34.7)	116 (38.7)	
	51-60	52 (34.7)	60 (40.0)	112 (37.3)	
	61-70	26 (17.3)	25 (16.7)	51 (17.0)	
	Median (Range)	52 (19–68)	52 (28–70)	52 (19-70)	ᵇ 0.569
Gender	Male	114 (76.0)	95 (63.3)	209 (69.7)	ᵃ 0.017
	Female	36 (24.0)	55 (36.7)	91 (30.3)	
Body Mass Index (kg/m^2^)	Normal	16 (10.7)	58 (38.7)	74 (24.7)	ᵃ <0.001
	Overweight	115 (76.7)	80 (53.3)	195 (65.0)	
	Obese	19 (12.7)	12 (8.0)	31 (10.3)	
	Median (Range)	27.25 (23.20-33.30)	25.70 (22.90-32.60)	26.5 (22.90-33.30)	ᵇ <0.001

**Figure 1:**
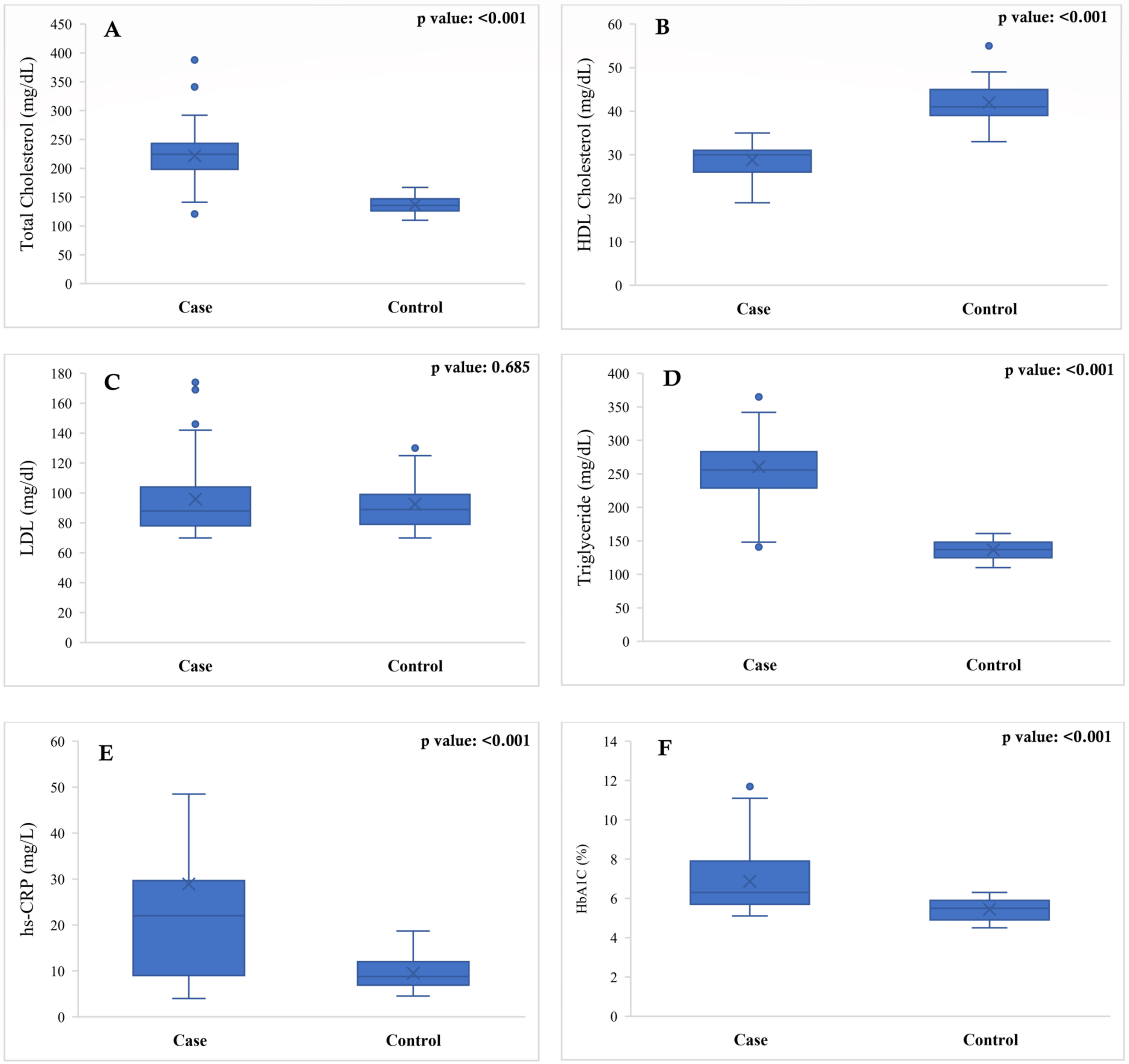
Comparison of serum (A) total cholesterol, (B) HDL, (C) LDL, (D) triglycerides, (E) hs-CRP, and (F) HbA1c between the case and control groups. LDL: low-density lipoprotein; HDL: high-density lipoprotein; Hs-CRP: high-sensitivity C-reactive protein; HbA1c: glycosylated hemoglobin The Mann-Whitney U test was done.

The laboratory profile showed marked biochemical differences between the case and control groups. Patients with CAD had significantly higher total cholesterol and triglyceride levels than controls, while high-density lipoprotein (HDL) cholesterol was substantially lower in the case group; all of these differences were highly statistically significant (p-value: <0.001), indicating a distinctly more atherogenic lipid pattern among cases. In contrast, low-density lipoprotein (LDL) cholesterol levels did not differ significantly between the two groups (p-value: 0.685). Inflammatory and metabolic markers also demonstrated clear separation: hs-CRP concentrations were markedly elevated in cases compared with controls (p-value: <0.001), supporting a strong association between systemic inflammation and CAD. Glycemic control was poorer in the case group, as reflected by significantly higher HbA1c values (p-value: <0.001). Overall, the findings indicate that CAD patients exhibited a more adverse lipid profile, higher inflammatory burden, and poorer glycemic status, while LDL cholesterol did not discriminate between the two groups (Figure 1).

**Figure 2:**
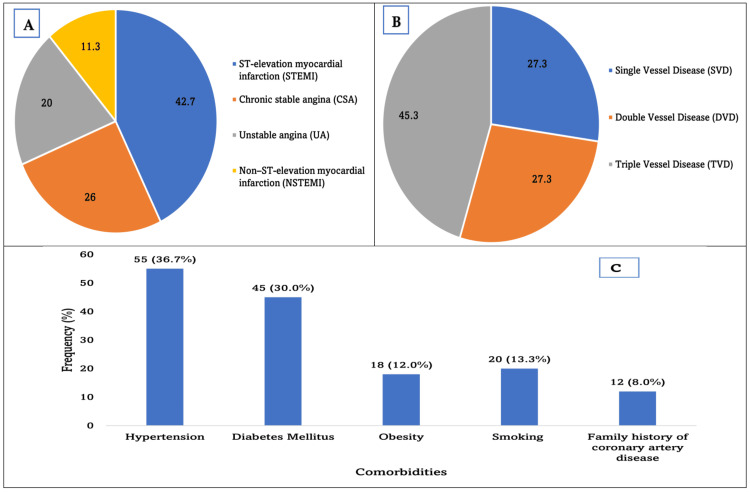
(A) Clinical presentation, (B) angiographic severity, and (C) comorbidities among the case group (n = 150)

Among the 150 patients in the case group, the most frequent clinical diagnosis was ST-elevation myocardial infarction (STEMI), which accounted for 42.7% (n=64) of the study population. This was followed by chronic stable angina (CSA) in 26.0% (n=39) of patients and unstable angina (UA) in 20.0% (n=30) of cases. Non-STEMI (NSTEMI) represented the smallest proportion, occurring in 11.3% (n=17) of participants. Assessment of CAD severity among the 150 cases revealed that triple-vessel disease was the most prevalent pattern, occurring in 45.3% (n=68) of patients. This was followed by double-vessel disease in 27.3% (n=41) and single-vessel disease in 27.3% (n=41) of the study population. These findings indicate that multi-vessel involvement was more common than single-vessel disease, with nearly half of the patients exhibiting severe coronary disease burden. Among the 150 patients in the case group, hypertension was the most common comorbidity, present in 36.7% (n=55) of participants. Diabetes mellitus was identified in 30.0% (n=45), while smoking and obesity were reported in 13.3% (n=20) and 12.0% (n=18), respectively. A family history of CAD was documented in 8.0% (n=12) cases. These findings demonstrate that hypertension and diabetes mellitus were the predominant comorbid conditions, indicating their importance as contributing cardiovascular risk factors within this cohort (Figure 2).

**Figure 3:**
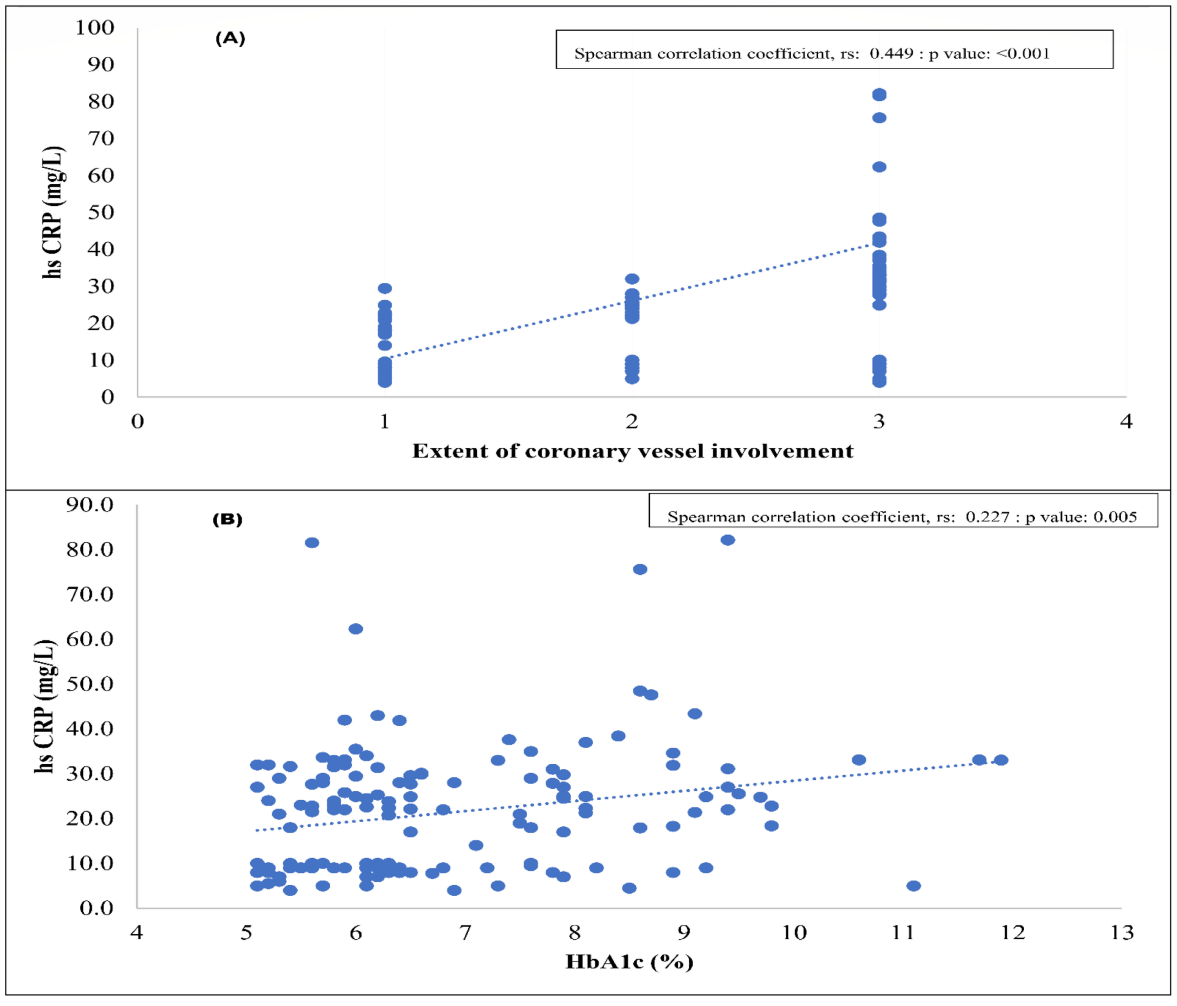
(A) Correlation between high-sensitivity C-reactive protein (hs-CRP) level and the extent of coronary vessel involvement; (B) Correlation between high-sensitivity C-reactive protein (hs-CRP) level and glycosylated hemoglobin (HbA1c) (n=150) Spearman correlation test was done.

Hs-CRP showed a weak positive correlation with HbA1c (r_s_ = 0.227, p-value: 0.005) and a moderate positive correlation with the extent of vessel involvement (r_s_ = 0.449, p-value: < 0.001). These findings indicate that higher levels of systemic inflammation were significantly associated with poorer glycemic control and greater severity of CAD (Figure 3).

**Figure 4:**
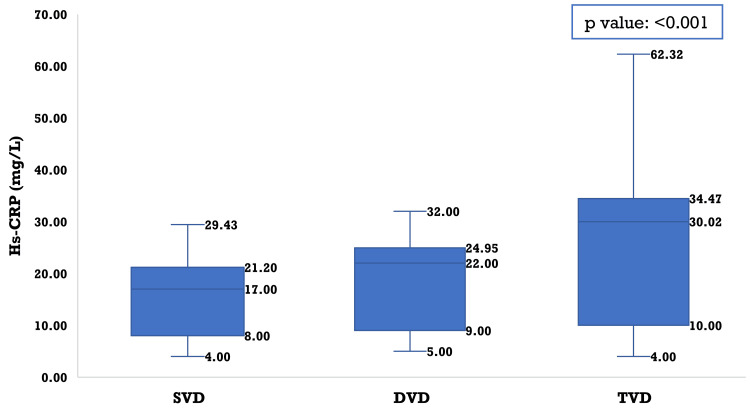
Distribution of high-sensitivity C-reactive protein (hs-CRP) according to the extent of coronary vessel involvement (SVD, DVD, and TVD) The Kruskal-Wallis test was done. SVD: single-vessel disease; DVD: double-vessel disease; TVD: triple-vessel disease

In contrast, hs-CRP demonstrated a strong and clinically meaningful gradient across severity groups (p-value: < 0.001). Patients with triple-vessel disease had markedly higher hs-CRP levels compared to single-vessel disease (p-value: < 0.001) and double-vessel disease (p-value: 0.002), indicating a robust association between systemic inflammation and increasing coronary vessel involvement. The difference between single-vessel disease and double-vessel disease was not significant (p-value: 0.268) (Figure 4).

**Figure 5:**
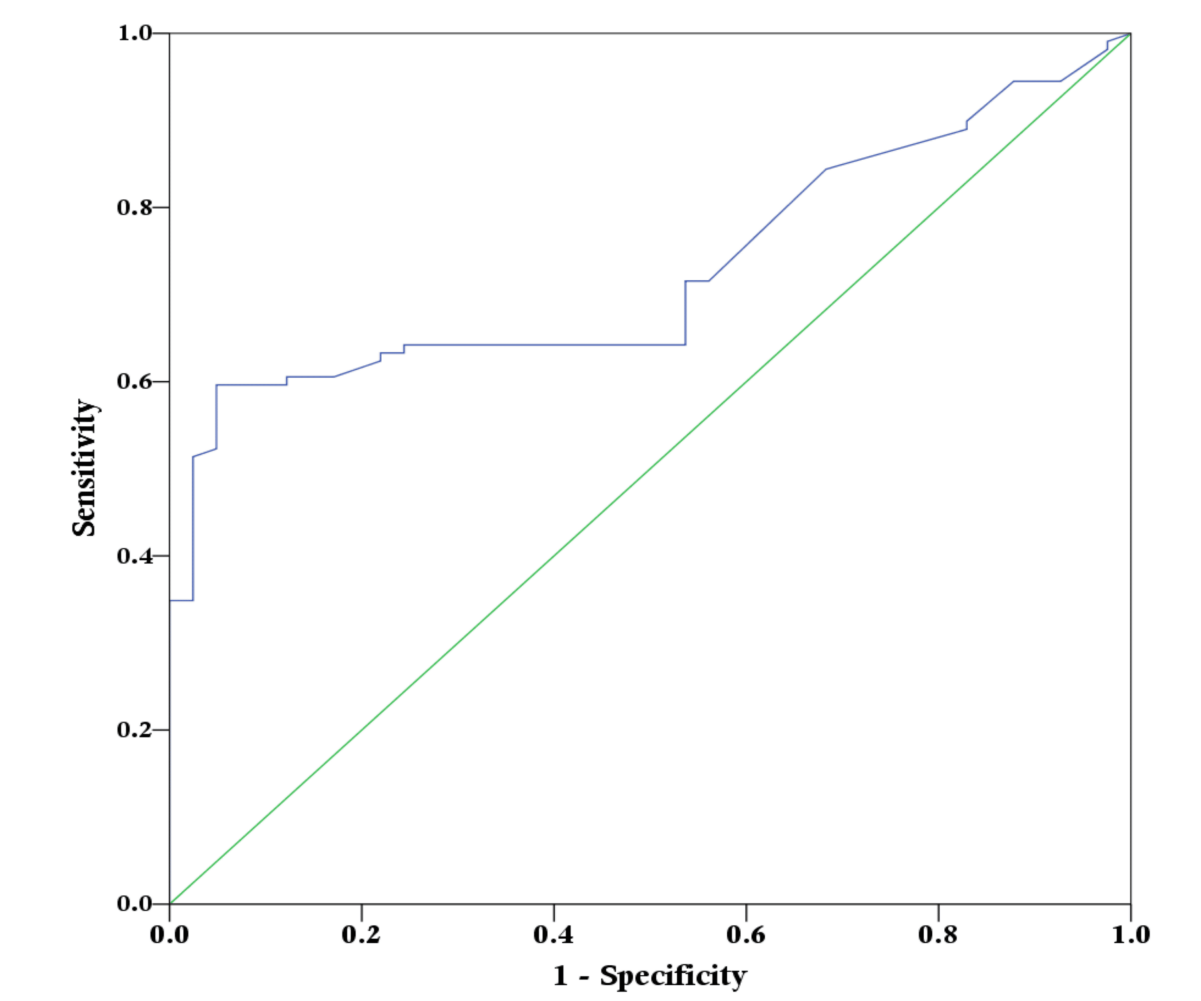
Receiver operator characteristics (ROC) curve of high-sensitivity C-reactive protein level to predict multi-vessel coronary artery disease

ROC curve analysis of Hs-CRP level to predict multi-vessel CAD, with an AUC value of 0.729 (95% CI: 0.651-0.808), which was statistically significant (p-value: <0.001) (Figure 5).

A cut-off value of hs-CRP ≥ 22.89 mg/L yielded the highest Youden index (0.5475), indicating an optimal balance between sensitivity and specificity for predicting multi-vessel CAD (Table 2).

**Table 2:** Determination of cut-off value of high-sensitivity C-reactive protein level to predict multi-vessel coronary artery disease with youden index PPV: positive predictive value; NPV: negative predictive value

Cut-off	Sensitivity	Specificity	PPV	NPV	Accuracy	Youden's Index
≥22.32	0.606	0.878	0.930	0.456	0.680	0.4836
≥22.89	0.596	0.951	0.970	0.470	0.693	0.5475
≥24.91	0.514	0.976	0.982	0.430	0.640	0.4894

At this hs-CRP ≥ 22.89 mg/L, the sensitivity was 59.6% (95% CI: 49.81%-68.92%) and the specificity was 95.1% (95% CI: 83.47%-99.40%). The overall diagnostic accuracy achieved with this cut-off was 69.3%. Furthermore, the positive predictive value and negative predictive value were 97.0% and 47.0%, respectively. These findings suggest that an hs-CRP level of ≥22.89 is highly specific and reasonably accurate for identifying patients with multi-vessel CAD, supporting its potential utility as a predictive marker, particularly for ruling in advanced coronary involvement (Table 3).

**Table 3:** Sensitivity, specificity, positive predictive value, negative predictive value, and accuracy gained by the derived cutoff of serum high-sensitivity C-reactive protein level with a 95% confidence interval for predicting multi-vessel coronary artery disease PPV: positive predictive value; NPV: negative predictive value

Statistic	Value	(95% Confidence Interval)	
		Lower	Upper
Sensitivity	59.63%	49.81%	68.92%
Specificity	95.12%	83.47%	99.40%
PPV	97.01%	89.29%	99.22%
NPV	46.99%	41.12%	52.94%
Accuracy	69.33%	61.29%	76.59%

## Discussion

The role of inflammation in the pathogenesis of CAD is well established; however, uncertainty remains regarding whether hs-CRP can reliably reflect both the presence and angiographic severity of disease, particularly in South Asian and resource-limited settings. In this context, the present study was undertaken to examine the relationship between hs-CRP and CAD burden, including differentiation between single- and multi-vessel involvement. Our findings demonstrate that hs-CRP levels were significantly higher in patients with CAD and increased progressively with the extent of vessel involvement, with acceptable discriminatory performance for identifying multivessel disease on ROC analysis. These results suggest that hs-CRP may serve as a practical adjunctive biomarker for pre-procedural risk stratification in routine clinical practice and may also provide a basis for further research targeting inflammatory pathways in the management of CAD.

In the present study, most participants were in their fifth decade of life, and males predominated, a pattern consistent with the well-recognized higher incidence of CAD among men, particularly between the fourth and seventh decades of life [15]. The higher proportion of CAD among males has also been reported from other Bangladeshi studies [16] and countries with an almost similar demographic profile [17]. In women, cardiovascular risk increases more gradually and rises sharply after the fifth and sixth decades, largely attributed to the decline in circulating estrogen levels following menopause, as estrogen exerts vasoprotective and anti-inflammatory effects that may mitigate atherosclerotic progression [15]. From a Bangladeshi perspective, sociocultural and health-system factors may further accentuate sex-based disparities, as women frequently delay seeking care, present with atypical symptoms, and experience lower diagnostic accuracy, leading to missed or late diagnoses and delays in initiating appropriate therapy. Consequently, women are less likely to undergo invasive coronary interventions such as percutaneous coronary intervention or coronary artery bypass grafting, which may partly explain their lower representation among angiographically confirmed cases in this cohort.

Patients with CAD in the present study exhibited a distinctly more adverse metabolic and inflammatory profile than controls, characterized by elevated total cholesterol and triglycerides, reduced HDL cholesterol, higher HbA1c, and substantially increased hs-CRP concentrations. These findings are consistent with established evidence linking dyslipidemia, insulin resistance, and chronic low-grade inflammation to atherosclerotic plaque development and progression [18]. The absence of a significant difference in LDL cholesterol between groups may reflect widespread statin exposure prior to angiography or population-specific lipid patterns, a phenomenon reported in several South Asian cohorts where triglyceride-rich lipoproteins and low HDL appear to play a particularly prominent role in cardiovascular risk [19].

In the present study, nearly half of the patients exhibited triple-vessel disease, indicating a substantial burden of advanced coronary involvement at the time of angiography. This proportion is considerably higher than that reported in another Bangladeshi cohort, where approximately one-fifth of CAD patients had triple-vessel disease, and also exceeds figures from comparative analyses in which about 28% of patients were found to have multi-vessel disease, even though Bangladeshi ethnicity itself was identified as an independent risk factor for extensive CAD [20,21]. Several factors may account for these discrepancies. Differences in study design, referral patterns, and inclusion criteria are likely contributors; as a tertiary-care-based investigation, the present cohort may have preferentially captured patients with more severe or symptomatic disease who were referred for invasive evaluation. Delayed presentation, a recognized issue in Bangladesh owing to limited access to specialized cardiac services, financial constraints, and low awareness of early symptoms, may further result in patients undergoing angiography at a more advanced stage of atherosclerosis. Atherosclerosis can develop slowly over many years and accelerate diffuse coronary involvement without causing symptoms until a major cardiovascular event occurs in patients with a longer duration of uncontrolled traditional risk factors such as diabetes mellitus, hypertension, smoking, central obesity, and dyslipidemia, which is also evident among the study participants with CAD [22]. The predominance of STEMI as the initial clinical presentation further suggests a high thrombotic and plaque-rupture burden, which is biologically plausible in the setting of heightened systemic inflammation. Importantly, acute myocardial infarction is an inflammatory condition and is known to elevate hs-CRP levels independent of the underlying atherosclerotic burden. Therefore, the higher hs-CRP levels observed in this study may partially reflect the acute-phase response rather than chronic inflammation alone.

A key contribution of the present study is the demonstration of a graded increase in hs-CRP across angiographic severity categories, with patients with triple-vessel disease exhibiting markedly higher levels than those with single- or double-vessel involvement. The moderate positive correlation between hs-CRP and vessel number supports the concept that inflammatory burden parallels anatomic disease extent. Accumulating evidence consistently supports the association between systemic inflammation and angiographic severity of CAD. Sharma (2023) demonstrated that elevated hs-CRP levels were significantly associated with an increasing number of diseased coronary vessels, while Rotty et al. (2023) reported a positive correlation between hs-CRP concentrations and the Gensini score, reinforcing the relationship between inflammatory burden and anatomical disease complexity [23,24]. Earlier, Gupta et al. (2013) documented a similar association in Indian patients with ACS, and Habib and Al Masri (2013) further showed that hs-CRP levels were significantly higher in patients with angiographically confirmed CAD compared to healthy controls and correlated with both disease presence and severity [25,26]. Collectively, these studies substantiate the role of hs-CRP as a marker of inflammatory activity associated with multi-vessel involvement and greater coronary complexity, with contemporary data from South Asian cohorts helping to clarify previously inconsistent findings. In contrast, Bouzidi et al. reported no significant association between hs-CRP and angiographic severity, a discrepancy that may be attributed to variations in patient risk profiles, baseline inflammatory status, sample size, or differences in clinical presentation and study methodology [27]. Indeed, individuals with apparently limited vessel involvement may still harbor long or multiple plaques, substantial luminal stenoses, elevated inflammatory markers, and a high burden of cardiometabolic risk factors. The multifactorial nature of coronary artery disease and heterogeneity in symptom severity and plaque morphology may therefore contribute to variability in observed relationships between hs-CRP and angiographic extent [28]. Nevertheless, the biological plausibility of this association remains strong, as hs-CRP reflects interleukin-6-mediated hepatic activation and is experimentally linked to endothelial dysfunction, up-regulation of vascular adhesion molecules, oxidative stress, and pro-thrombotic states, processes that collectively promote diffuse plaque formation and instability.

Taken together, these pathophysiological links provide a strong rationale for exploring hs-CRP not only as a marker of inflammatory activity but also as a practical clinical tool for anticipating the anatomic burden of coronary disease before angiographic assessment, a premise that is supported by the present ROC-based diagnostic performance analysis. The ROC analysis in the present study demonstrated that hs-CRP has fair discriminatory ability for predicting multi-vessel CAD, with an AUC of 0.729. The derived cut-off value of ≥22.89 mg/L showed very high specificity and positive predictive value, indicating that markedly elevated hs-CRP levels are particularly useful for identifying patients who are likely to harbor advanced coronary involvement. Although sensitivity and negative predictive value were more modest, this performance profile suggests that hs-CRP may function primarily as a “rule-in” marker, helping clinicians prioritize patients for early invasive evaluation in settings where catheterization laboratory access is limited. These findings are broadly consistent with previous investigations that reported higher discriminatory performance for hs-CRP in predicting angiographic severity. One study documented an AUC of 0.869 (95% CI 0.721-0.872; p < 0.001), with an optimal cut-off value of 2.78 mg/L yielding 80.2% sensitivity and 85% specificity for severe CAD [11], while another reported an even higher AUC of 0.966 (95% CI 0.943-0.989), achieving 100% sensitivity and 80.34% specificity for predicting extensive disease [29]. Differences in optimal cut-off thresholds and diagnostic accuracy across studies likely reflect variation in patient populations, baseline inflammatory status, prevalence of ACS, assay characteristics, and definitions of angiographic severity, underscoring the importance of population-specific calibration when applying hs-CRP-based risk stratification in clinical practice. Given that hs-CRP testing is inexpensive, widely available, and easily incorporated into routine laboratory panels, its integration with traditional risk factors could enhance pre-angiographic risk stratification, especially in ACS or high-risk stable patients.

From a clinical perspective, these findings support the concept of residual inflammatory risk in CAD and underscore the potential relevance of targeting inflammation alongside lipid-lowering and glycemic control. Patients with markedly elevated hs-CRP and suspected CAD may represent a subgroup with diffuse disease who could benefit from intensified preventive strategies, closer surveillance, and aggressive modification of cardiometabolic risk factors. In addition, therapeutic strategies aimed at reducing inflammatory markers may be required and could potentially provide additional clinical benefit in selected patients. In low- and middle-income countries such as Bangladesh, where diagnostic delays are common, such simple biomarkers may have particular utility in guiding triage and therapeutic decision-making.

Limitations

Several limitations should be considered when interpreting the findings of this study. First, the cross-sectional design limits the ability to establish causal relationships. In addition, hs-CRP was measured at a single time point, which may not reflect temporal variations in inflammatory status and precludes assessment of its potential role as a baseline biomarker for longitudinal monitoring of disease progression or prediction of future cardiovascular events. Although strict exclusion criteria were applied to minimize confounding from overt inflammatory and systemic conditions, this resulted in a relatively selected study population and may limit the generalizability of the findings to routine clinical settings where multiple comorbidities are common. Furthermore, subclinical or unrecognized sources of inflammation cannot be entirely excluded. The use of purposive sampling and recruitment from tertiary-care centers may also have introduced selection bias toward patients with more advanced or symptomatic disease. In addition, angiographic severity was assessed based on the number of involved vessels rather than more detailed scoring systems such as SYNTAX or Gensini scores, which could provide a more comprehensive assessment of coronary disease complexity. Future prospective studies incorporating longitudinal follow-up, multivariable analysis, and detailed angiographic scoring are warranted to validate these findings.

## Conclusions

hs-CRP was significantly higher in patients with CAD and was able to discriminate the presence of disease. hs-CRP levels also increased with angiographic severity and were higher in patients with multivessel involvement, especially triple-vessel disease. A moderate positive correlation was found between hs-CRP and the number of diseased vessels. ROC analysis further demonstrated that hs-CRP has fair discriminatory ability for predicting multivessel coronary disease, with a cut-off value of ≥22.89 mg/L providing very high specificity and positive predictive value, supporting its role as a useful “rule-in” biomarker for advanced coronary involvement. These findings reinforce the close relationship between systemic inflammation and diffuse atherosclerotic burden and highlight the potential clinical utility of hs-CRP as an adjunct to traditional risk factors for pre-angiographic risk stratification, particularly in resource-limited settings such as Bangladesh. Collectively, the results provide a rationale for further research exploring inflammatory pathways and their role in the assessment and management of CAD.
